# Practical manual of pharmacology

**Published:** 2008-08

**Authors:** Chetna Desai

**Affiliations:** M.D. (Pharmacology), CMCL FAIMER Fellow, Associate Professor in Pharmacology, B. J. Medical College, Ahmedabad - 380 016, Gujarat, India. E-mail: chetna99@gmail.com

Pharmacology for undergraduate students has witnessed major (and at times radical) changes in the past few years. The medical student, a potential prescriber, should be capable of keeping pace with the continuing developments in pharmacological management of various diseases. Hence, there is a shift in the conventional approach of acquisition of knowledge, practical skills and developing attitudes in students, which train them to be a rational prescriber. Recommendations now focus on clinical pharmacy and pharmacology, problem based exercises, pharmacovigilance and computer assisted learning. These changes have rendered the existing practical journals and manuals either obsolete or incomplete. A need has, therefore, been felt for a manual that documents why and how a practical should be conducted. This book has fulfilled the need and, in fact, gone beyond merely fulfilling the need. The book has been written keeping in mind the desired paradigm shift in the practical curriculum for undergraduate medical students.

The chapters have been organized in sections like clinical pharmacy, experimental pharmacology and clinical pharmacology mainly for clarity and convenience. It covers all the feasible exercises in pharmacology that can be carried out by most institutions, with minimal resources. A useful feature of this book is the ample use of examples, which will help not only the trainee teachers but also the students. Each chapter is neatly presented with brevity and accuracy. The objectives of learning are clearly defined, although they could well have been stated at the beginning of the chapter. The clear and relevant illustrations can substitute the lack of materials, which may be the case at some centers.

The section on clinical pharmacy, while preserving the importance of dispensing, meticulously describes the commonly used dosage forms and routes of drug administration. This section also makes a generous use of illustrations. The section on experimental pharmacology describes those experiments that are necessary for the student to understand the fundamental mechanisms of drug action. The author also emphasizes ethics in animal experimentation and suggests ways of substituting these with computer simulation exercises. The clinical pharmacology section emphasizes on prescription writing through problem based learning, with a liberal use of examples. The chapters on prescription audit and problem based drug interactions are likely to develop a critical and analytical approach in students. Other relevant chapters include those on pharmacokinetics, dosage calculations, Adverse Drug Reactions (ADR) monitoring and therapeutic drug monitoring, critical appraisal of promotional literature and evaluation of drug formulations.

An appendix on proposed revised curriculum and teaching schedule offers guidance to teachers who wish to implement them in their departments. The Essential Drugs List of India (2003) and the Adverse Drug Reactions Monitoring Form are also provided for ready reference. It is indeed a meticulously written book; a ready reckoner for teachers and a useful manual for the undergraduate students.

